# Cortical activity and functional organisation during ocular pursuit is affected by concurrent upper limb movement

**DOI:** 10.1038/s41598-026-52172-9

**Published:** 2026-05-18

**Authors:** Lénaïc Borot, Ruth Ogden, Simon J. Bennett

**Affiliations:** 1 School of Sport and Exercise Sciences, Faculty of Science, Liverpool John Moores University, Liverpool, UK; 2 School of Psychology, Faculty of Health, Liverpool John Moores University, Liverpool, UK

**Keywords:** Psychology, Human behaviour, Motor control, Oculomotor system

## Abstract

Tracking a moving object with the eyes involves sensory-motor and cognitive processes, and is supported by a wide network of cortical areas. We investigated if cortical activity and network organisation in young adults are influenced by the availability of retinal input when pursuing a moving object, and whether this is modulated by extra-retinal input from concurrent upper limb movement. As expected, we found a decrease in average eye velocity, and increase in saccadic displacement, when the moving object was occluded, as well as a general facilitatory effect of oculo-manual tracking. We also found decreased activity in prefrontal and frontal cortex during oculo-manual compared to ocular tracking when the moving object was occluded. Following a short period of practice in the oculo-manual condition without occlusion, there was an increase in activity in prefrontal, parietal and visual cortex during ocular tracking. These findings could indicate how extra-retinal input during oculo-manual tracking reduces the need for attentional and predictive processes to extrapolate and pursue the occluded object. This is an important step in better understanding impaired oculo-manual coordination (e.g., age-related decline), potentially informing the development of more effective tasks for differential diagnosis and rehabilitation.

## Introduction

Smooth pursuit eye movement (SPEM) is known to involve a wide range of cortical regions^1–3^, with activity modulated by factors that affect trajectory predictability. For example, an increased bilateral activation of prefrontal (DLPFC), frontal (medial FEF, SEF), pre-motor and parietal (SPL, IPS) cortex has been found in trials where the pursuit object was transiently occluded compared to where it remained visible^4^. It was subsequently suggested^5^ that these areas were activated as part of a compensatory mechanism that attempts to maintain SPEM by predicting the occluded object trajectory. Extending upon this work, it was reported^6^ that bilateral FEF activation was evident irrespective of a moving object’s visibility, whereas bilateral DLPFC activation increased when the object was occluded, as well as when trajectory predictability decreased due to the absence of additional cues (No Trace, Partial Trace, Full Trace). There were also stronger inter-hemispheric and intra-hemispheric correlations for FEF and DLPFC when the object was occluded. It was suggested that although a functional interaction exists between FEF and DLPFC whenever participants pursue a moving object, these areas make distinct contributions to oculomotor control depending on the task demands and associated requirement for higher-order cognitive processes.

In our recent work^7^, we showed that PFC (DLPFC, MPFC) activity and network organisation were modified when SPEM was performed with concurrent upper limb movement. We suggested that afference and/or efference from upper limb movement could have provided extra-retinal information on the occluded object trajectory (for a model and behavioural data see^8^, which modulated the predictive processes operating in PFC. However, facilitation of SPEM and the influence on PFC activity by concurrent upper limb movement were less than expected, in part due to the use of discrete, short duration externally-generated object motion. Indeed, much of the previous work showing facilitation of SPEM by concurrent upper limb movement^9,10^ required participants to pursue cyclical object motion (i.e., triangular or sine wave) over a duration of several seconds. This provided greater opportunity for sharing of information between the ocular and motor control systems, which improved following several minutes of practice^10^. In addition, we did not consider the contribution of PPC and FEF, both of which are active during smooth pursuit of a visible object^3,5^ and play an important role in eye-hand coordination^11^. For example, these regions are part of the dorsal attention network (DAN), which is involved in overt and covert spatial attention, and thus linked with the fronto-parietal network (FPN), which is involved in cognitive processes such as working memory during goal-directed tasks^12,13^.

In the current study, we investigated cortical activity and network organisation within a number of cortical areas known to be involved during SPEM, as well as the modulatory effect of extra-retinal input provided by concurrent upper limb movement. Functional near-infrared spectroscopy (fNIRS: 24 × 24 optode array) was used to image regions of prefrontal (MPFC, DLPFC), frontal (FEF) and parietal (IPL, SPL) and visual cortex (VC) while participants pursued (eyes alone or eyes and upper limb) a sinusoidal object motion that was either continuously visible or transiently occluded (predictable location and duration). Measurements were taken before and after a short practice period in which participants pursued a continuously visible object with eyes and upper limb. It was expected that smooth pursuit during occlusion would be enhanced by access to extra-retinal input from concurrent upper limb movement, although not to the extent that average eye velocity would match object velocity, even after a period of practice. Given the functional coupling between smooth pursuit and saccadic eye movements during object tracking, we also anticipated a concomitant increase in saccadic eye displacement as the oculomotor system attempted to match total eye displacement to object displacement. Moreover, we expected that the oculo-manual facilitation during occlusion would offset the demand on attentional and predictive processes, particularly following a short period of practice, which would then be reflected by changes in both cortical activity and network organisation.

## Results

### Behavioural data

As will be described in more detail below, average eye velocity, saccadic displacement and total eye displacement differed with the availability of retinal input from the moving object and/or extraretinal input from the effectors (see Table 1). There was also some indication that average eye velocity and saccadic displacement differed between pre-test and post-test, but this effect was more pronounced in the hand velocity data.

**Table 1:** Summary of statistical results for eye velocity, saccadic displacement, total eye displacement and hand velocity. Columns show: Effector, Dependent Variable, Model, Effect, Chisq, degrees of freedom (df), P-values (raw and adjusted), conditional and marginal R2.

Effector	Dependant variable	Model	Effect	Chisq	Df	*P* value	Adjusted *P* value	R2C	R2M
Eye	Velocity	Data ~ Occlusion * Tracking + Occlusion * Test + (1 | Subject)	Occlusion	5081.86	1	< 0.001 ***	< 0.001 ***	0.9	0.86
			Tracking	94.02	1	< 0.001 ***	< 0.001 ***		
			Test	0.06	1	0.81346	0.9307		
			Occlusion: Tracking	5.26	1	< 0.05 *	< 0.05 *		
			Occlusion: Test	5.98	1	< 0.05 *	< 0.05 *		
			Tracking: Test	0.12	1	0.734	0.931		
			Occlusion: Tracking: Test	1.49	1	0.222	0.381		
	Saccadic displacement	Data ~ Occlusion * Tracking + Occlusion * Test + (1 | Subject)	Occlusion	894.64	1	< 0.001 ***	< 0.001 ***	0.66	0.53
			Tracking	77.53	1	< 0.001 ***	< 0.001 ***		
			Test	0.01	1	0.93	0.93		
			Occlusion: Tracking	2.52	1	0.11	0.21		
			Occlusion: Test	1.27	1	0.26	0.42		
			Tracking: Test	0.07	1	0.80	0.93		
			Occlusion: Tracking: Test	0.04	1	0.85	0.93		
	Total eye displacement	Data ~ Occlusion * Tracking * Test + (1 | Subject)	Occlusion	15.74	1	< 0.001 ***	< 0.001 ***	0.21	0.03
			Tracking	7.05	1	< 0.01 **	< 0.05 *		
			Test	2.61	1	0.106	0.208		
			Occlusion: Tracking	0.01	1	0.919	0.931		
			Occlusion: Test	0.79	1	0.373	0.560		
			Tracking: Test	0.34	1	0.558	0.787		
			Occlusion: Tracking: Test	0.01	1	0.915	0.931		
Hand	Velocity	Data ~ Occlusion * Test + (1 | Subject)	Occlusion	37.11	1	< 0.001 ***	< 0.001 ***	0.41	0.18
			Test	40.05	1	< 0.001 ***	< 0.001 ***		
			Occlusion: Test	26.69	1	< 0.001 ***	< 0.001 ***		

Significant values are in [bold].

#### Occlusion × tracking

**Fig. 1:**
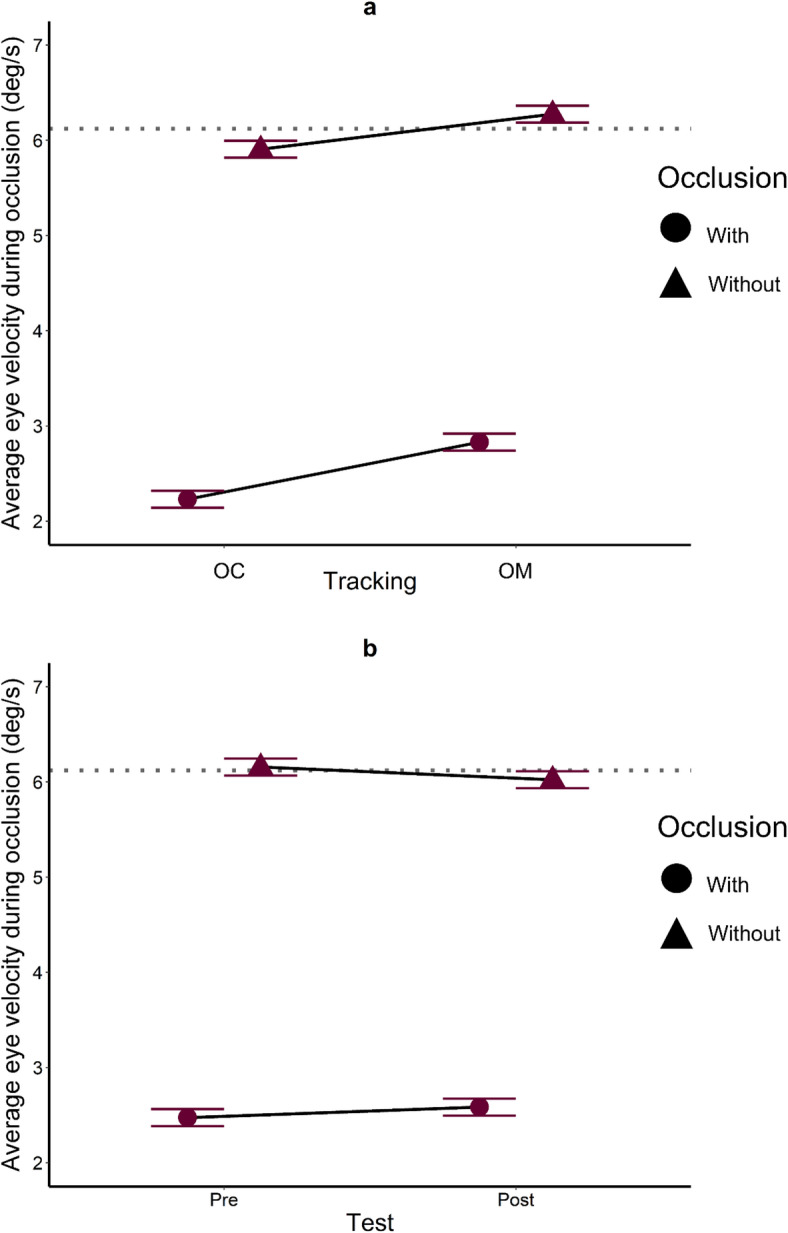
Average eye velocity during occlusion (**a**: Occlusion × Tracking interaction; **b**: Occlusion × Test interaction). The grey dotted line corresponds to average object velocity during occlusion. Large markers represent the estimated marginal means, with error bars indicating the standard errors.

As can be seen in Fig. 1, eye velocity was lower during OC and OM tracking in trials with occlusion (2.23 deg/s, 2.83 deg/s) than without occlusion (5.91 deg/s, 6.27 deg/s). Moreover, in both trials with and without occlusion, eye velocity was lower in the OC than OM tracking condition. For saccadic eye displacement, the interaction was not significant, but there were significant main effects of Occlusion and Tracking. Saccadic eye displacement was higher during OC (4.12 deg) than OM (2.96 deg) tracking, and in trials with occlusion (5.51 deg) than without occlusion (1.57 deg). For total eye displacement, there was also only a main effect of Occlusion and Tracking. Total eye displacement was higher during OC (8.00 deg) than OM (7.83 deg) tracking, and in trials with occlusion (8.04 deg) than without occlusion (7.79 deg).

#### Occlusion × test

**Fig. 2:**
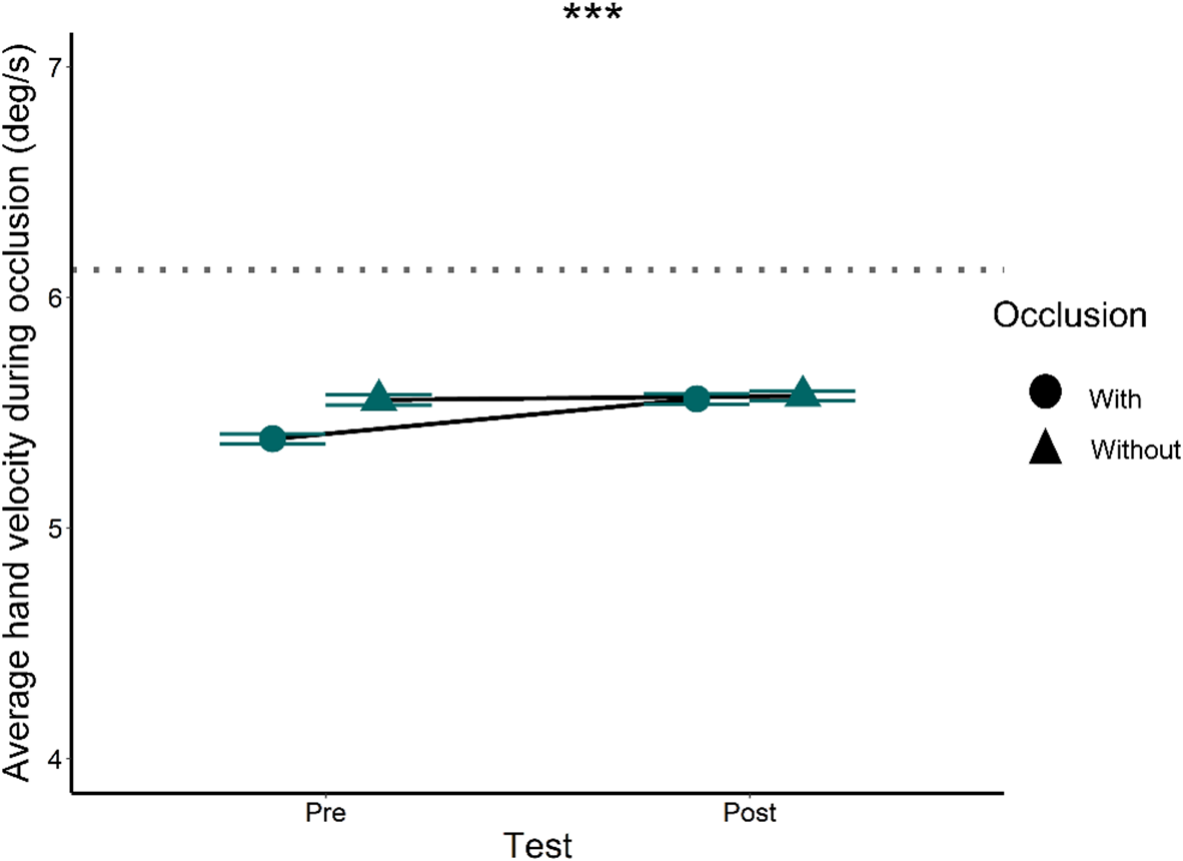
Average hand velocity during occlusion (significant Occlusion x Test interaction). The grey dotted line corresponds to average object velocity during occlusion. Large markers represent the estimated marginal means, with error bars indicating the standard errors.

Eye velocity in trials with occlusion was lower than trials without occlusion at both pre-test (2.47 deg/s; 6.16 deg/s) and post-test (2.58 deg/s; 6.02 deg/s). For saccadic displacement and total eye displacement, there were no significant interaction or main effects involving Test. As can be seen in Fig. 2, hand velocity in trials with occlusion increased from pre-test (5.39 deg/s) to post-test (5.56 deg/s), but there was no change in trials without occlusion (5.56 deg/s; 5.57 deg/s). As a consequence, although hand velocity was lower at pre-test in trials with than without occlusion, there was no difference at post-test.

### Neuroimaging data

#### Cortical activity

**Fig. 3:**
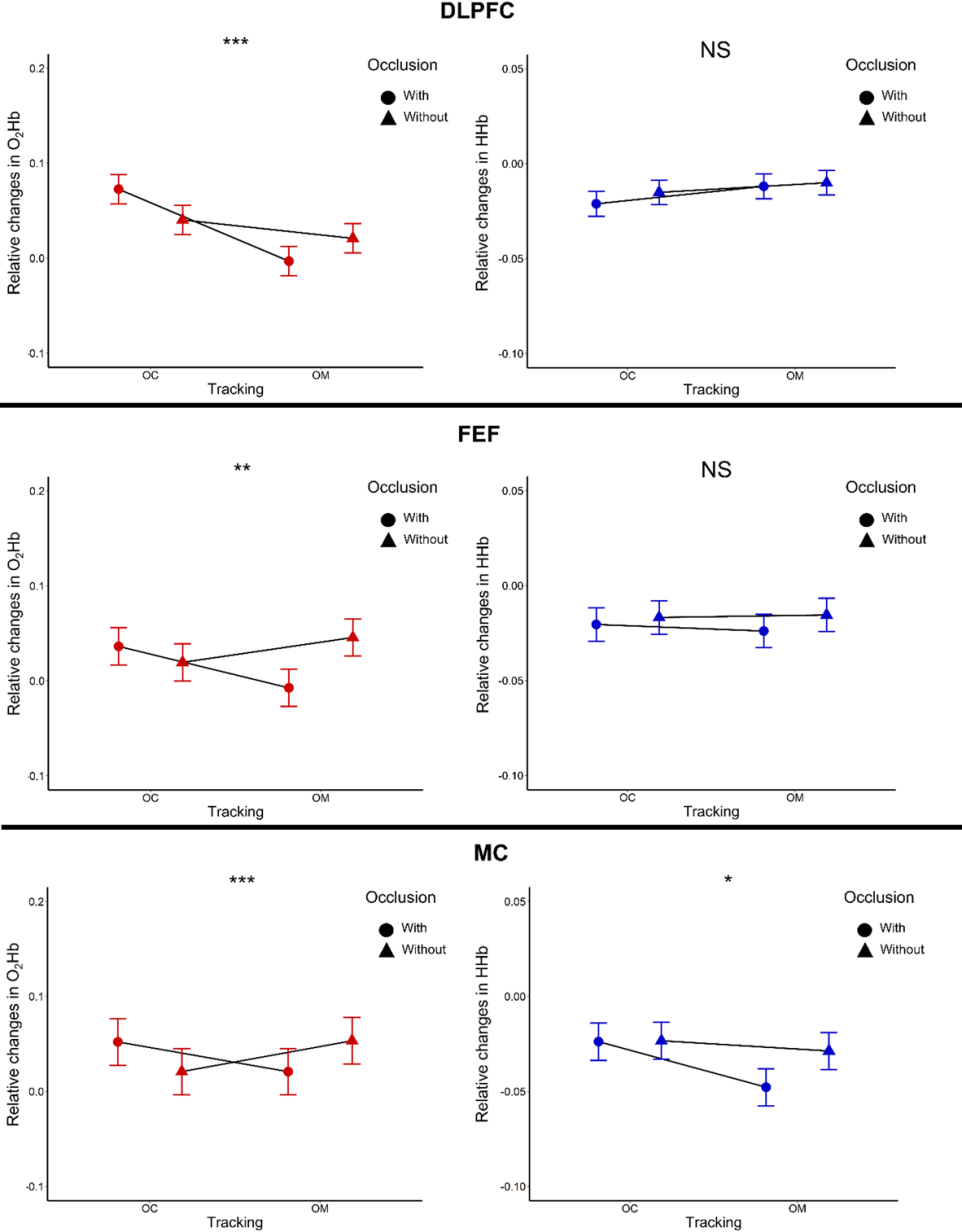
Occlusion × Tracking interaction for O_2_HB (red) and HHb (blue). Large markers represent the estimated marginal means, with error bars indicating the standard errors. NS *p* > 0.05, * *p* < 0.05, ** *p* < 0.01, *** *p* < 0.001.

**Fig. 4:**
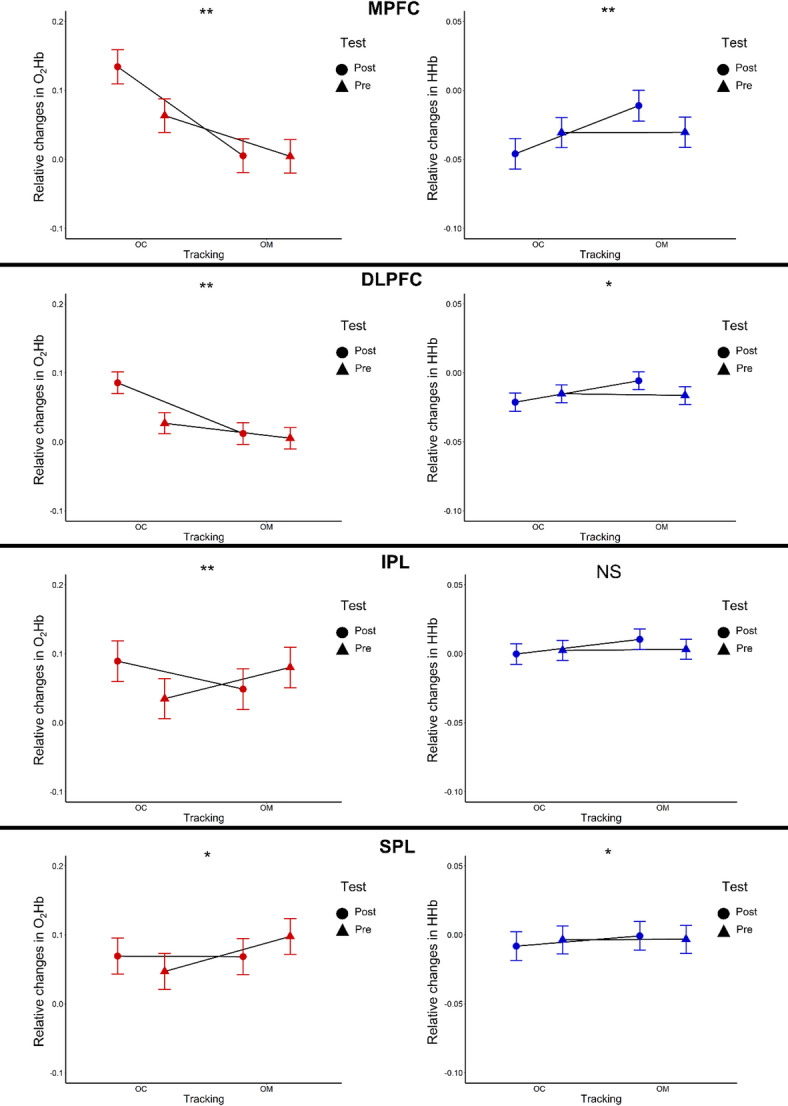
Tracking × Test interaction for O_2_HB (red) and HHb (blue). Large markers represent the estimated marginal means, with error bars indicating the standard errors. NS *p* > 0.05, * *p* < 0.05, ** *p* < 0.01, *** *p* < 0.001.

Across all regions measured, but particularly in PFC through to MC, cortical activity (O_2_Hb and HHb) differed as a function of the availability of retinal input and/or the effectors used to track the moving object (see Table 2). As can be seen in Fig. 3 (Occlusion × Tracking) and Fig. 4 (Tracking x Test), the pattern of effects was similar in each of these regions, although pairwise comparisons did not always indicate significance.

**Table 2:** Summary of statistical results for O_2_Hb and HHb for each ROI. Columns show: ROI, Chromophore, Model, Effect, Chisq, degrees of freedom (df), P-values (raw and adjusted), conditional and marginal R2.

ROI	Chromophore	Model	Effect	Chisq	Df	P value	Adjusted P value	R2C	R2M
MPFC	O_2_Hb	Data ~ Occlusion*Test*Tracking+ Hemisphere (1 | Subject) + (1 | Subject: Channel)	Occlusion	1.45	1	0.23	0.37	0.15	0.034
			Test	10.85	1	< 0.001 ***	< 0.01 **		
			Tracking	74.51	1	< 0.001 ***	< 0.001 ***		
			Hemisphere	3.06	1	0.08	0.18		
			Occlusion: Test	0.06	1	0.81	0.84		
			Occlusion: Tracking	4.95	1	< 0.05 *	0.08		
			Test: Tracking	10.99	1	< 0.001 ***	< 0.01 **		
			Occlusion: Test: Tracking	1.97	1	0.16	0.31		
DLPFC	O_2_Hb	Data ~ Occlusion*Test*Tracking+ Hemisphere (1 | Subject) + (1 | Subject: Channel)	Occlusion	0.39	1	0.53	0.69	0.11	0.02
			Test	21.00	1	< 0.001 ***	< 0.001 ***		
			Tracking	42.42	1	< 0.001 ***	< 0.001 ***		
			Hemisphere	6.96	1	< 0.01 **	< 0.05 *		
			Occlusion: Test	0.28	1	0.59	0.74		
			Occlusion: Tracking	16.24	1	< 0.001 ***	< 0.001 ***		
			Test: Tracking	13.78	1	< 0.001 ***	< 0.01 **		
			Occlusion: Test: Tracking	0.06	1	0.81	0.84		
FEF	O_2_Hb	Data ~ Occlusion*Test*Tracking+ Hemisphere (1 | Subject) + (1 | Subject: Channel)	Occlusion	2.89	1	0.09	0.19	0.16	0.01
			Test	3.04	1	0.08	0.18		
			Tracking	0.58	1	0.45	0.59		
			Hemisphere	1.84	1	0.17	0.33		
			Occlusion: Test	1.43	1	0.23	0.37		
			Occlusion: Tracking	11.45	1	< 0.001 ***	< 0.01 **		
			Test: Tracking	0.79	1	0.37	0.51		
			Occlusion: Test: Tracking	0.18	1	0.67	0.80		
MC	O_2_Hb	Data ~ Occlusion*Test*Tracking+ Hemisphere (1 | Subject) + (1 | Subject: Channel)	Occlusion	0.00	1	0.97	0.97	0.21	0.01
			Test	1.18	1	0.28	0.43		
			Tracking	0.04	1	0.84	0.86		
			Hemisphere	0.22	1	0.64	0.78		
			Occlusion: Test	2.42	1	0.12	0.24		
			Occlusion: Tracking	15.61	1	< 0.001 ***	< 0.001 ***		
			Test: Tracking	0.12	1	0.73	0.81		
			Occlusion: Test: Tracking	3.52	1	0.06	0.15		
IPL	O_2_Hb	Data ~ Occlusion*Test*Tracking+ Hemisphere (1 | Subject) + (1 | Subject: Channel)	Occlusion	1.53	1	0.22	0.37	0.18	0.01
			Test	1.00	1	0.32	0.46		
			Tracking	0.16	1	0.69	0.80		
			Hemisphere	10.87	1	< 0.001 ***	< 0.01 **		
			Occlusion: Test	4.61	1	< 0.05 *	0.09		
			Occlusion: Tracking	3.91	1	< 0.05 *	0.12		
			Test: Tracking	13.41	1	< 0.001 ***	< 0.01 **		
			Occlusion: Test: Tracking	2.57	1	0.11	0.23		
SPL	O_2_Hb	Data ~ Occlusion*Test*Tracking+ Hemisphere (1 | Subject) + (1 | Subject: Channel)	Occlusion	0.07	1	0.80	0.84	0.22	0.01
			Test	0.07	1	0.79	0.84		
			Tracking	6.24	1	< 0.05 *	< 0.05 *		
			Hemisphere	5.85	1	< 0.05 *	< 0.05 *		
			Occlusion: Test	6.92	1	< 0.01 **	< 0.05 *		
			Occlusion: Tracking	1.73	1	0.19	0.33		
			Test: Tracking	5.82	1	< 0.05 *	< 0.05 *		
			Occlusion: Test: Tracking	0.13	1	0.72	0.81		
VC	O_2_Hb	Data ~ Occlusion*Test*Tracking+ Hemisphere (1 | Subject) + (1 | Subject: Channel)	Occlusion	1.79	1	0.18	0.33	0.20	0.01
			Test	0.99	1	0.32	0.46		
			Tracking	19.92	1	< 0.001 ***	< 0.001 ***		
			Hemisphere	0.37	1	0.54	0.69		
			Occlusion: Test	1.16	1	0.28	0.43		
			Occlusion: Tracking	4.00	1	< 0.05 *	0.12		
			Test: Tracking	11.72	1	< 0.001 ***	< 0.01 **		
			Occlusion: Test: Tracking	0.82	1	0.37	0.51		
MPFC	HHb	Data ~ Occlusion*Test*Tracking+ Hemisphere (1 | Subject) + (1 | Subject: Channel)	Occlusion	6.00	1	< 0.05 *	0.06	0.18	0.01
			Test	0.15	1	0.70	0.76		
			Tracking	12.99	1	< 0.001 ***	< 0.01 **		
			Hemisphere	0.53	1	0.47	0.65		
			Occlusion: Test	1.14	1	0.29	0.50		
			Occlusion: Tracking	5.85	1	< 0.05 *	0.06		
			Test: Tracking	14.34	1	< 0.001 ***	< 0.01 **		
			Occlusion: Test: Tracking	0.06	1	0.81	0.88		
DLPFC	HHb	Data ~ Occlusion*Test*Tracking+ Hemisphere (1 | Subject) + (1 | Subject: Channel)	Occlusion	2.08	1	0.15	0.30	0.16	0.01
			Test	0.88	1	0.35	0.59		
			Tracking	6.38	1	< 0.05 *	< 0.05 *		
			Hemisphere	1.50	1	0.22	0.41		
			Occlusion: Test	0.74	1	0.39	0.62		
			Occlusion: Tracking	0.30	1	0.59	0.71		
			Test: Tracking	10.41	1	< 0.01 **	< 0.05 *		
			Occlusion: Test: Tracking	18.19	1	< 0.001 ***	< 0.001 ***		
FEF	HHb	Data ~ Occlusion*Test*Tracking+ Hemisphere (1 | Subject) + (1 | Subject: Channel)	Occlusion	2.63	1	0.10	0.23	0.26	0.01
			Test	3.66	1	0.06	0.17		
			Tracking	0.19	1	0.66	0.76		
			Hemisphere	0.51	1	0.48	0.65		
			Occlusion: Test	0.43	1	0.51	0.65		
			Occlusion: Tracking	0.66	1	0.42	0.65		
			Test: Tracking	3.62	1	0.06	0.17		
			Occlusion: Test: Tracking	9.89	1	< 0.01 **	< 0.05 *		
MC	HHb	Data ~ Occlusion*Test*Tracking+ Hemisphere (1 | Subject) + (1 | Subject: Channel)	Occlusion	8.79	1	< 0.01 **	< 0.05 *	0.30	0.01
			Test	3.09	1	0.08	0.19		
			Tracking	20.05	1	< 0.001 ***	< 0.001 ***		
			Hemisphere	0.80	1	0.37	0.61		
			Occlusion: Test	2.98	1	0.08	0.20		
			Occlusion: Tracking	8.20	1	< 0.01 **	< 0.05 *		
			Test: Tracking	8.61	1	< 0.01 **	< 0.05 *		
			Occlusion: Test: Tracking	4.15	1	< 0.05 *	0.14		
IPL	HHb	Data ~ Occlusion*Test*Tracking+ Hemisphere (1 | Subject) + (1 | Subject: Channel)	Occlusion	0.44	1	0.51	0.65	0.17	0.01
			Test	0.57	1	0.45	0.65		
			Tracking	2.39	1	0.12	0.25		
			Hemisphere	0.28	1	0.60	0.71		
			Occlusion: Test	15.44	1	< 0.001 ***	< 0.01 **		
			Occlusion: Tracking	0.16	1	0.69	0.76		
			Test: Tracking	1.87	1	0.17	0.33		
			Occlusion: Test: Tracking	2.81	1	0.09	0.21		
SPL	HHb	Data ~ Occlusion*Test*Tracking+ Hemisphere (1 | Subject) + (1 | Channel)	Occlusion	0.03	1	0.87	0.89	0.03	0.01
			Test	0.40	1	0.53	0.66		
			Tracking	17.57	1	< 0.001 ***	< 0.001 ***		
			Hemisphere	0.05	1	0.83	0.88		
			Occlusion: Test	3.57	1	0.06	0.17		
			Occlusion: Tracking	0.26	1	0.61	0.71		
			Test: Tracking	9.71	1	< 0.01 **	< 0.05 *		
			Occlusion: Test: Tracking	3.21	1	0.07	0.19		
VC	HHb	Data ~ Occlusion*Test*Tracking+ Hemisphere (1 | Subject) + (1 | Subject: Channel)	Occlusion	0.47	1	0.49	0.65	0.15	0.00
			Test	0.04	1	0.85	0.88		
			Tracking	0.56	1	0.46	0.65		
			Hemisphere	3.30	1	0.07	0.19		
			Occlusion: Test	0.00	1	0.96	0.96		
			Occlusion: Tracking	1.28	1	0.26	0.46		
			Test: Tracking	0.49	1	0.49	0.65		
			Occlusion: Test: Tracking	4.65	1	< 0.05 *	0.11		

Significant values are in [bold].

#### O_2_Hb

##### Occlusion × tracking

In DLPFC and FEF, mean O_2_Hb was lower during OM (-0.00321e-06; -0.00742e-06) than OC tracking (0.07264e-06; 0.03631e-06), but only in trials with occlusion. In addition, mean O_2_Hb in DLPFC during OC tracking was higher in trials with than without occlusion (0.07264e-06; 0.04027e-06), whereas mean O_2_Hb in FEF during OM tracking was lower in trials with than without occlusion (-0.00742 e-06; 0.04563e-06). In MC, mean O_2_Hb was higher during OM than OC tracking in trials without occlusion (0.0534e-06; 0.0209e-06), and lower during OM than OC tracking in trials with occlusion (0.0208e-06; 0.0521e-06). It was also higher during OC tracking in trials with than without occlusion (0.0521e-06; 0.0209e-06), and lower during OM tracking in trials with than without occlusion (0.0208e-06; 0.0534e-06).

##### Tracking × test

Mean O_2_Hb in MPFC was lower at both pre-test and post-test during OM (0.00452e-06; 0.00527e-06) than OC tracking (0.06345e-06; 0.13430e-06), and increased from pre-test to post-test during OC tracking. In DLPFC, mean O_2_Hb at post-test was lower during OM than OC tracking (0.01220 e-06; 0.08580e-06), and also increased from pre-test to post-test during OC tracking (0.02711e-06; 0.08580e-06). In IPL, mean O_2_Hb at pre-test was higher during OM than OC tracking (0.0803e-06; 0.0349e-06), and increased from pre-test to post-test during OC tracking (0.0349e-06; 0.0892e-06). In SPL, mean O_2_Hb at pre-test was higher during OM than OC tracking (0.0978e-06; 0.0693e-06). In VC, mean O_2_Hb at post-test was higher during OC than OM tracking (0.07923e-06; -0.03146e-06), and also increased from pre-test to post-test during OC tracking (0.01876e-06; 0.07923e-06).

#### HHb

##### Occlusion × tracking

In MC, mean HHb was lower during OM than OC tracking in trials with occlusion (-0.0478e-06; -0.0238e-06), and lower during OM tracking in trials with than without occlusion (-0.0478e-06; -0.0287 e-06).

##### Tracking × test

Mean HHb in MPFC was higher during OM than OC tracking condition at post-test (-0.0110e-06; -0.0459e-06), and increased from pre-test to post-test during OM tracking (-0.0303e-06; -0.0110e-06). Mean HHb in MC increased from pre-test (-0.0464e-06) to post-test (-0.0301e-06) during OM tracking. It was also lower during OM than OC tracking at pre-test (-0.0464e-06; -0.0216e-06). In SPL, mean HHb was higher during OM (0.01518e-06) than OC (-0.01439e-06) tracking at post-test.

##### Occlusion × tracking × test

Mean HHb in DLPFC was higher during OM than OC tracking in trials with occlusion, but only at post-test (-0.00233e-06; -0.03072e-06). Mean HHb in DLPFC during OC tracking was also lower in trials with occlusion than without occlusion (-0.03072e-06; -0.01168e-06), but again only at post-test. Finally, mean HHb in trials with occlusion was lower at post-test than pre-test during OC tracking (-0.03072e-06; -0.01153e-06), and higher at post-test-test than pre-test during OM tracking (-0.00233e-06; -0.02157e-6). In FEF, mean HHb during OM tracking increased from pre-test (-0.03849e-06) to post-test (-0.00935e-06) in trials with occlusion.

#### Network organisation

**Fig. 5:**
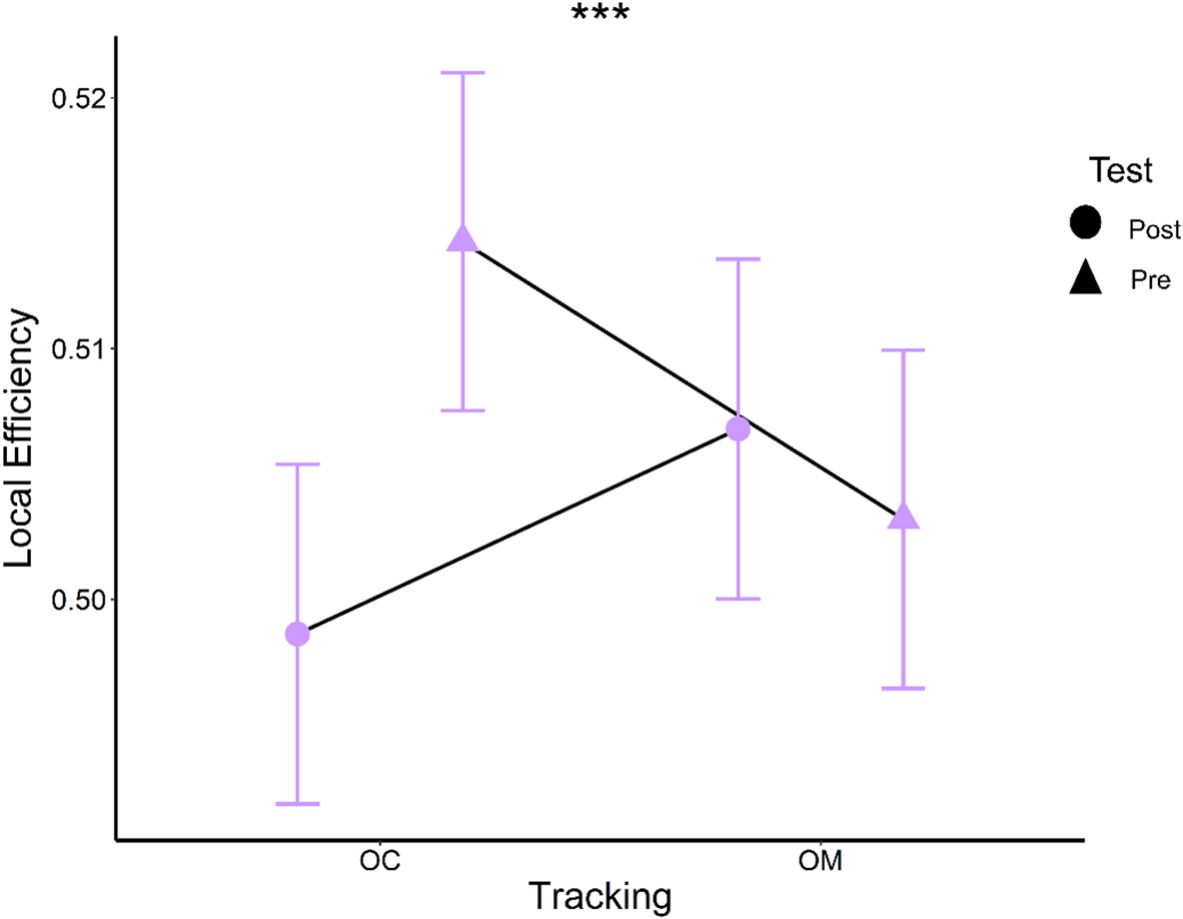
Local efficiency (significant tracking × test interaction). Large markers represent the estimated marginal means, with error bars indicating the standard errors.

Similar to the measures of behaviour and cortical activity, local efficiency differed between pre-test and post-test, although this was influenced by the effectors used to track the moving object (see Fig. 5). Specifically, during OC tracking local efficiency was higher at pre-test (0.514) than post-test (0.499). Also, local efficiency was higher during OC (0.514) than OM (0.503) tracking at pre-test, but lower during OC (0.499) than OM (0.507) tracking at post-test. There were no significant effects found for global efficiency.

## Discussion

Studies of SPEM in young, human adults have shown task-dependent cortical activity within a number of regions, with the magnitude and correlation (interhemispheric and intrahemispheric) modulated by the availability of retinal input^4,6^. It has been suggested that these effects can be explained by an increased need for attentional and predictive processes to compensate for the inevitable reduction in SPEM during occlusion^5^. Here, our primary aim was to examine if the availability of extra-retinal input from concurrent upper limb movement (i.e., afference and/or efference) influences cortical activity and network organisation when pursuing a moving object that was either continuously visible or transiently occluded with predictable timing and duration. In accord with recent discussion on how to improve the reliability and repeatability of fNIRS studies^14,15^, we report findings for both chromophores (O_2_Hb and HHb) as an indirect measure of cortical activity. However, we focus our discussion on the changes in O_2_Hb as they are usually of higher amplitude and less sensitive to noise than changes in HHb^16^, and thus more reflective of task-dependent cortical activity.

As expected, our behavioural measures of eye movement were influenced by the availability of retinal and/or extra-retinal input from the upper limb. Average smooth eye velocity decreased, and saccadic eye displacement increased, in trials where the moving object was occluded^17,18^. There was also clear evidence of oculo-manual facilitation, with increased average smooth eye velocity, and decreased saccadic eye displacement, when pursuing the moving object with the eyes and upper limb compared to eyes alone. This oculo-manual facilitation was evident irrespective of whether the moving object remained visible or was occluded. That said, average smooth eye velocity during occlusion was much lower than average object velocity, resulting in gain of 0.46, compared to 1.03 when the object remained visible^9^. Still, when combined with the saccadic response, total eye displacement was well matched to object displacement, resulting on average in a small overshoot of less than 0.5 deg. An increase in smooth eye velocity coupled with a decrease in saccades during oculo-manual tracking is a characteristic of visually-guided eye-hand coordination, where participants attempt to maintain a more stable gaze and minimize saccadic suppression. However, such a response is unnecessary during occlusion, where there is no visual feedback from the moving object or upper limb. Instead, it is more likely that the observed oculo-manual facilitation during occlusion is a result of extra-retinal inputs from limb afference and/or efference improving the predictive processes required to extrapolate and pursue the occluded object trajectory.

Consistent with the importance of retinal input to oculomotor control, there was little effect of the practice period^19^. Only hand velocity in trials with occlusion increased from pre-test to post-test, becoming closer to the average object velocity (see Fig. 2). This increase was only small, but is consistent with the fact that the hand, unlike the eyes, can be moved voluntarily at or near object velocity in the absence of retinal input. A subsidiary analysis in which centred hand velocity was included as a covariate, along with the fixed and random effects in the original linear mixed model, indicated that extra-retinal input from hand movement did indeed influence smooth pursuit (Table 4). Hand velocity significantly predicted average smooth eye velocity (β = 0.64, *p* = 0.012), although as expected occlusion had a much larger effect (β = − 1.69, *p* < 0.0001). Importantly, none of the interactions involving hand velocity were significant, indicating that this eye-hand coordination was stable across occlusion conditions and between pre-test and post-test.

There was also evidence across all cortical regions that activity (O_2_Hb) was influenced by the availability of retinal input and/or extra-retinal input. Consistent with prefrontal cortex being involved in attentional and predictive processes, we found increased activity in DLPFC during ocular tracking when the moving object was occluded compared to when it remained visible^4,5^. Activity in FEF during ocular tracking was similar irrespective of object visibility^6^. This can be interpreted in line with previous work^3^ that showed FEF is involved in attentional smooth pursuit object tracking independent of object frequency, and thus task difficulty (see also^20^. They are not, however, consistent with the finding of increased bilateral activation of mesial FEF, as well as left lateral FEF, in trials where the pursuit object was transiently occluded compared to when it remained visible^4^. The increased activation in left FEF was subsequently found to be negatively correlated with reduced eye velocity during occlusion^5^. We cannot be certain why there are differences in FEF activation reported between these studies, but it has been suggested that they could in part be explained by the use of different object motion characteristics and analysis procedures^3^. For example, the use of short duration, constant velocity ramps^4,5^ may have been less predictable than longer duration sine wave motion^3^, which was also used in the current study. Still, despite these differences in cortical activity between the aforementioned studies, there is a developing consensus about DLPFC and FEF being part of a compensatory mechanism that attempts to maintain SPEM of a moving object’s trajectory during occlusion: for a behavioural model see^21,22^. Finally, we also found evidence of increased activity in motor cortex during ocular tracking when the moving object was occluded compared to when it remained visible. This may reflect a limitation in the spatial resolution of our NIRS setup, which used 24 × 24 optodes at pre-determined locations (81 long distance channels and 8 short distance channels) within the NIRS cap (10-5 coordinate system). This resulted in the ROI specified as motor cortex (see Fig. 7) actually encompassing both primary motor cortex (BA4) and pre-motor cortex (BA6). Notably, pre-motor cortex exhibits increased activity during pursuit of an occluded object because it is involved in the coordination of oculomotor commands with attentional and predictive processes^4^.

A further notable finding was the decreased activity in prefrontal (DLPFC) and frontal (FEF) cortex during oculo-manual compared to ocular tracking when the object was occluded. Based on the previously reported role of prefrontal cortex described above, it would seem that extra-retinal inputs from limb afference and/or efference reduced the need for attentional and predictive processes to extrapolate and pursue the occluded object trajectory. For motor cortex, there was decreased activity during oculo-manual compared to ocular tracking when the object was occluded, which could again be explained by the role of pre-motor cortex in attentional and predictive processes. There was also decreased activity in motor cortex during oculo-manual tracking when the moving object was occluded compared to when it remained visible. The latter finding could be expected given the reduced need for visual guidance of upper limb movement during occlusion, and an associated reduction in the control of visual attention. We have previously shown in a similar pursuit task that there are fewer corrections in upper limb movement when the object is occluded compared to when it remains visible^23^, which is consistent with the suggestion that control of the upper limb during occlusion is based on a comparison of sensorimotor signals (i.e., limb afference and efference) to an internal representation of object motion^24^.

As for practice effects, a consistent finding across prefrontal (MPFC, DLPFC) parietal (IPL) and visual cortex (VC) was an increase in activity from pre-test to post-test during ocular tracking. In prefrontal (MPFC, DLPFC) cortex, this resulted in higher activity at post-test during ocular than oculo-manual tracking, whereas in parietal cortex (IPL, SPL) it minimised any difference between the two tracking conditions that existed at pre-test. There was also evidence that cortical organisation changed following the period of practice. Specifically, local efficiency (measure of network segregation) decreased from pre-test to post-test during ocular tracking when the object was occluded. As a consequence, while local efficiency was higher during ocular than oculo-manual tracking at pre-test, it was lower at post-test. Together, our finding of similar practice‑related changes across multiple ROIs and network-level organisation could be indicative of how a short period of oculo-manual practice influences subsequent cortical activity during ocular tracking. In this respect, it is relevant to note that although relatively short in duration, the opportunity to practice the oculo-manual task for approx. 6 min in the current study is comparable to previous work with a similar sinusoidal tracking task, where it was reported that human adults only require a few minutes of training to achieve an accurate and stable oculo-manual control^10^. Also, the observed practice effects in the current study were specific to the ocular tracking condition rather than being consistent across all conditions. This would not be expected if the change in cortical activity was a result of a general practice effect associated with familiarity or arousal. Taken together, we suggest that these findings are broadly consistent with previous work^11^ indicating the role of a distributed parieto-frontal network in eye-hand coordination, where the contribution from specific cortical areas changes as a function of task demand and experience. However, further studies with longer practice periods, additional manipulations such as transfer tasks, and a retention test will be needed to clarify and extend these initial observations.

Unlike some previous work^5^, we did not seek to directly assess the potential link between cortical activity in specific ROIs and behaviour. Our approach was based on the premise that brain networks are characterised by complex integration and segregation, such that behaviour typically reflects coordinated activity across distributed regions rather than isolated contributions from single ROIs. To capture this network-level organisation, we did consider global efficiency, which reflects information exchange between multiple ROIs. However, there were no changes in this graph metric across any of the independent factors. A possible explanation could be that global efficiency is more likely to change within subnetworks, such as the associative system (e.g. fronto-parietal network) and sensory-motor system (e.g. visual network, motor network), rather than across the entire network formed by the 50 pairs of NIRS channels in the current study. Some evidence consistent with this position can be found in previous work^6^, where it was reported that inter-hemispheric and intra-hemispheric correlations between DLPFC and FEF were generally higher when pursuing an occluded vs. continuously visible object. To provide further insight on this issue, future work could consider using alternative multivariate or network‑based approaches to examine how haemodynamic changes relate to behaviour in object pursuit tasks.

Although there were changes in both chromophores for all ROIs, these were not always significant and thus consistent with a theoretical pattern in which there is an increase in O_2_Hb and a concurrent but weaker decrease in HHb. This is a well-known phenomenon in fNIRS^25^, but it is not entirely clear why this theoretical pattern is not always present. Some explanations include inter-subject variability^26^, task difficulty^27^, or variability between processing pipelines (e.g. model based vs. model free hemodynamic response estimation)^28^. As stated above, it could also in part be related to the fact that changes in O_2_Hb are usually of higher amplitude and less sensitive to noise than changes in HHb^16^. In this respect, it is important to note that we used several methods in the current study to check signal quality, as well as control measures including baseline correction to subtract resting state haemodynamic activity, regression of short-distance channels from long-distance channels to reduce extracerebral noise, and preprocessing steps to improve signal quality^25^. Therefore, we suggest the results for O_2_Hb are more likely to represent task-evoked changes in the haemodynamic response rather than a false positive as a consequence of a confounding factor. Here, it is also relevant to comment that although some of marginal means were negative for O_2_Hb, and positive for HHb, these were calculated relative to the steady-state hemodynamic activity during baseline, and importantly were not significantly different from 0 in all but one case. Therefore, rather than being considered as decreased activity in this ROI, they are more likely to indicate similar activity as baseline.

In summary, the results of the current study indicate that oculo-manual tracking influences SPEM, cortical activity and network organisation, consistent with extra-retinal input reducing the demand on attentional and predictive processes when pursuing an occluded object trajectory. These findings from young, healthy adults provide an important first step to subsequently understand how cortical activity and organisation during oculo-manual tracking is affected by factors such as normal aging or neurological conditions, potentially informing the development of more effective tasks for differential diagnosis and rehabilitation.

## Materials and methods

### Participants

Twenty-eight participants (16 males/ 12 females) from the University staff and student population volunteered to take part in the study (mean age of 26.54 ± 5.79 years). Given the difficulties associated with a priori sample size calculation with linear mixed-effects models, our sample size was based on sample sizes used in comparable studies. All participants were right-handed and self-declared with normal or corrected vision and no neurological impairment. All participants provided a written informed consent to participate in the study. The study was approved by the Liverpool John Moores University Research Ethics Committee (20/SPS/014) and was conducted in accordance standards of the Declaration of Helsinki.

### Task and procedure

Participants came to the laboratory on a single occasion for approximately one hour. Having being given verbal and written instructions on the experimental protocol, participants were invited to sit on a height-adjustable chair at a worktop, after which the cap and optodes of the NIRS neuroimaging system (NIRSport2, NIRX) was placed on their head. To minimize potential crosstalk between the fNIRS system and the IR light from the EyeLink illuminator, a piece of black material was used to cover the optodes. Also, the lights in the laboratory were extinguished during the experiment. Next, participants were asked to place their chin and forehead on a support, which ensured their eyes were located 915 mm away from a 24-inch LCD screen (ViewPixx EEG) with 1280 × 1024 pixels resolution and 100 Hz refresh rate. An EyeLink 1000 with remote optics was located beneath the lower edge of the LCD screen and used to record eye gaze at 250 Hz. Participants gaze location was calibrated relative to the LCD screen using a nine-point grid prior to each block of trials.

**Fig. 6:**
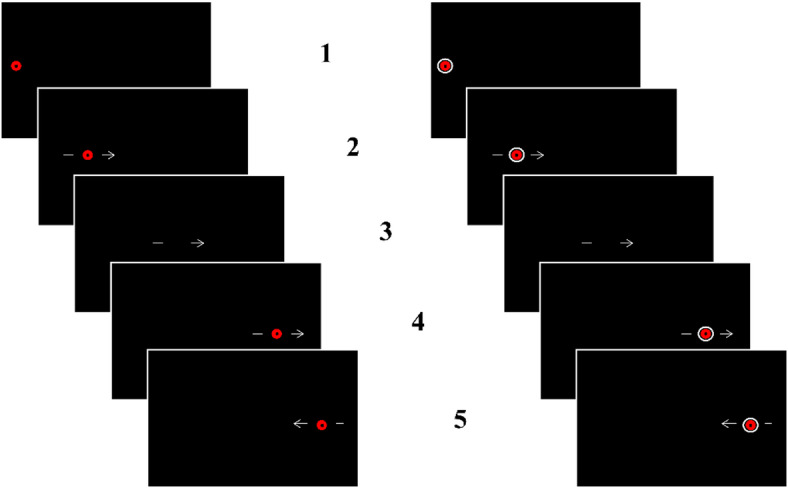
Schematic diagram showing the timeline of a trial for the ocular (left) and oculo-manual conditions (right). In the latter, a grey annulus line representing hand movement of the stylus on the tablet was drawn on the screen. Nb. White arrow depicting direction of object motion was not visible to participants. Panel 3 represents the occlusion, during which the object and anulus were not visible to participants.

Having completed the initial set-up the experiment commenced, which comprised two test phases (pre and post), separated by a short period of practice. Participants were asked to pursue a red circular object (0.5 degrees diameter with a black dot at its centre), which moved horizontally against a black background on the LCD screen in accord with a sine wave (20 deg amplitude and 0.1 Hz frequency) for 3.5 cycles (35s trial duration). In the pre-test and post-test, the moving object was either visible throughout the entire trial or was occluded (not during the first cycle) for 1250ms (Fig. 6, panel 3). The occlusion was aligned to the mid-point (screen centre) of a cycle as the object moved from left to right and from right to left of the screen (i.e., 5 occlusion events per trial, see Fig. 6). Participants were asked to pursue the moving object as accurately as they could with eyes alone (ocular– OC) or with eyes and hand (oculo-manual-OM). This resulted in four conditions (OC and OM with and without object occlusion), in which three trials were performed in a randomised order, resulting in a total of 12 trials. In practice, participants performed 10 trials in the OM condition without occlusion. Hand movement in the OM condition was recorded while participants moved a hand-held stylus on a Wacom A3 wide digitising tablet (250 Hz sampling rate). This provided real-time input on the horizontal position of the hand-held stylus, which was used to draw a grey anulus of 0.8 degrees diameter on the LCD screen (Fig. 6, right panels). Participants were instructed to keep the anulus surrounding the moving object as accurately as they could. The hand movement was performed at low frequency over a relatively small amplitude (20 cm), which combined with the comfortable and stable seating position was intended to minimize artefact in the fNIRS signals coming from head and upper body movement (see below for additional processing steps).

All trials started with 6s fixation, during which a white cross was displayed in the centre of the screen, and ended with a 30s rest period during which the screen was blank. During the last 3s second of fixation in the oculo-manual condition, the white annulus representing the hand-held stylus was displayed surrounding the fixation cross to inform participants that the next trial would involve manual tracking. Generation of the visual stimuli, recording of data from the Wacom digitising tablet and synchronisation with the EyeLink 1000 and NIRSport2 was achieved using the Cogent Toolbox in Matlab^®^ (MATLAB R2013b, The MathWorks, USA).

**Fig. 7:**
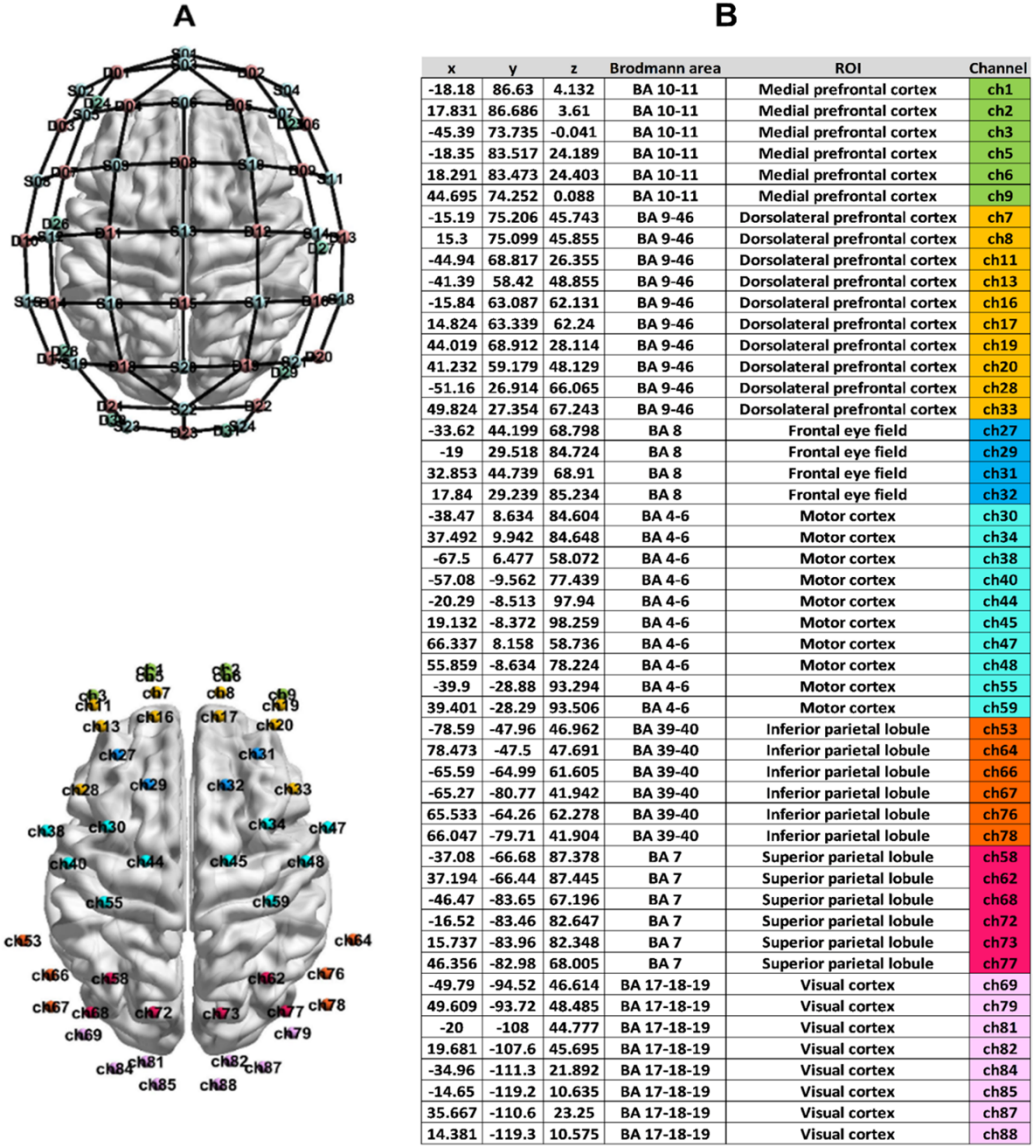
(**A**) Top: Representation of the 24 × 24 full optode organisation (emitters = light red dots; receivers = light blue dots) and channels (black edges) generated using BrainNet Viewer toolbox^30^. Bottom: Representation of channels included in each ROI (one colour per ROI). (**B**) MNI coordinates for each channel included within an ROI, as well as the Brodmann area covered by the channel identified using NFRI function^28^. In the right of the table, the channels included in an ROI can be identified by a colour assigned to each ROI.

Changes in O_2_Hb and HHb were quantified with functional near infrared spectroscopy (NIRSport2), using two wavelengths (760 nm and 850 nm) at a sampling rate of 6.8 Hz. Organisation of the 24 × 24 optodes was made using NIRsite software based on the 10-5 coordinate system, and resulted in a total of 81 long distance channels and 8 short distance channels (Fig. 7). To define regions of interest (ROIs), Brodmann areas covered by each channel were computed using the NFRI function^29^, which used the MNI (Montreal Neurological Institute) coordinates of the optode array reported by the manufacturer software. From these, we selected 7 ROIs in each hemisphere, comprised from 50 long distance channels (Fig. 7).

### Data preprocessing

*Eye and hand movement*: Horizontal eye position (relative to display reference system) and eye velocity (relative to head reference system) signals were exported using the Eyelink parser software. The software also identified and labelled saccades and blinks in the horizontal eye position. The criterion for saccade identification was a velocity threshold of 30 deg/s, acceleration threshold of 8000 deg/s^2^, and a motion threshold of 0.15 deg. Blinks were identified when the pupil in the camera could not be reliably determined, such as when it was distorted by eyelid occlusion. Using routines written in Matlab^®^ (MATLAB 2020b, The MathWorks, USA), the identified saccades and blinks were removed from the eye velocity trace, along with 5 additional data points before and after each event^18^. The resulting gaps due to saccades were replaced by linear interpolation based on steady-state smooth eye velocity surrounding the event (i.e., average of 5 data points). A further thresholding pass was performed to identify and remove without replacement any remaining eye velocity exceeding 15 deg/s. The desaccaded eye velocity data were processed with a zero-phase, low-pass filter (i.e., moving average filter with a 30 frame window semi-length: nanmoving_average by Carlos Vargas). As saccades during object tracking are typically of small amplitude, and thus short duration lasting only a few tens of milliseconds, linear interpolation takes place over a small number of samples, producing a velocity time series that approximates well the underlying smooth pursuit profile.

Using synchronisation signals from the stimulus generation routine (i.e., TTL), smooth eye velocity for each trial was identified. Average eye velocity during the five intervals (i.e., 1250ms) corresponding to an occlusion (i.e., same interval in trials without occlusion) was then calculated. Saccadic displacement occurring during these intervals was also calculated by summing the individual saccade amplitudes. To evaluate how well the combined smooth and saccadic response tracked the target, total eye displacement was calculated as the difference between eye position at the start and end of the 1250 ms interval.

Hand position data from the tablet was processed using custom-written routines in Matlab^®^ (MATLAB 2020b, The MathWorks, USA). Horizontal position data were processed with a zero-phase, low-pass filter (i.e., moving average filter with a 5 frame window semi-length: nanmoving_average) after which hand velocity was derived by applying a 3-point central difference calculation to the position data. Average hand velocity in each trial was calculated over the same intervals as smooth eye velocity. Finally, any velocity data that was subsequently found to exceed the group mean ± 3SDs was replaced with NaN. Such data were deemed to be indictive of participants not performing the task as instructed, and thus classified as outliers.

*Neuroimaging*: The first step of fNIRS preprocessing was to minimize the impact of signal noise on the subsequent data analysis. For this purpose, a consensus-based approach was applied to the raw data extracted from the Aurora software (2021.9). Three methods proposed in the literature to assess the signal quality were used. The first involved observation of the power spectrum density of the O_2_Hb signals for each channel for each participant, where the presence of a cardiac rhythm in the signal (peak around 1 Hz) indicates good contact between the scalp and optodes^31^. The second method used the coefficient of variation on O_2_Hb, with a maximal threshold of 15% used to define a channel as being of insufficient quality. The third method involved the application of QT-nirs^32^ with the following parameters: window: 3s; overlap: no; qualityThreshold = 0.75; sciThreshold = 0.7; pspThreshold = 0.1. Two channels were automatically excluded as the source-detector distance was too long. Channels not identified as being of good quality by at least 2 from 3 of the quality control methods were excluded from the subsequent analysis. Seven participants classified as having more than 33% excluded channels, or more than two ROIs without any good quality channels, were excluded from the fNIRS analysis. In addition, 2 participants only had data of sufficient quality at pre-test, but were retained for subsequent processing and analysis, with their post-test data coded as missing.

Data were next processed using functions from the Homer2 toolbox^33^. Raw data extracted from the Aurora software (2021.9) was converted to optical density, after which the following two methods were applied to reduce possible head motion artifacts as recommended in^34^: (1) moving standard deviation and spline interpolation^35^ using parameters: SDTresh = 20, AMPTresh = 0.5, tMotion = 0.5s, tMask = 2s and *p* = 0.99; (2) wavelet-based signal decomposition^36^ with iqr = 1.5. The optical density time series were next converted into concentrations of O_2_Hb and HHb using the modified Beer-Lambert law, with a differential pathlength factor depending on the age of the participant^37^. To limit the presence of physiological artifacts in the data, a high (0.009 Hz) and low pass (0.1 Hz) Butterworth zero phase digital filter (order 4) was applied. The signal from short distance channels (*n* = 8) was then regressed to the long-distance channels (NB. the short distance channels were regressed to long distance channels from the closest ROI).

Time series of O_2_Hb and HHb were extracted for each trial using synchronisation signals generated by the stimulus generation routine (i.e., TTL), and then baseline corrected. Working backward from the onset of target motion, there were 33s with no task‑related stimulation (i.e., first 3s of fixation with only a cross visible, plus 30s rest period). The final 3s of fixation differed between OM and OC conditions, and were excluded to avoid introducing condition‑specific bias into the baseline. From the 33s interval, we used the first 3s of the fixation together with 17s from the inter‑trial interval (20s total) to compute a stable baseline representative of resting state activity. The remaining 13 s of the inter-trial interval were deemed long enough to ensure sufficient recovery of the hemodynamic response before the next trial began.

The first trial of each pre-test and post-test was excluded from further analysis. Separately for O_2_Hb and HHb, the respective time series from channels within each ROI were averaged (see Fig. 7), after which the average concentration was extracted from the entire 35s interval of object motion. For measures of efficiency, graphs metrics (see below) per participant per trial were calculated by first detrending and then calculating partial Pearson correlations between the O_2_Hb time series for all pairs of channels. The resulting 50-by-50 partial correlation matrices (channel by channel from each ROI) were next subjected to z Fisher transformation, with all negative connections then set to zero. From the weighted positive matrices, local and global efficiency were extracted using functions implemented in Brain Connectivity Toolbox^38^. We chose to set negative correlations to zero rather than taking the absolute value, as this is a more conservative approach that avoids the assumption that anti-correlated activity supports network efficiency in the same way as positively correlated activity.

### Statistics

To ensure consistency in constructing the mixed-effects models, we followed a principled approach in which the specific fixed and random structures were adapted to reflect the factorial design applicable for each dependent variable. The criteria for model selection, optimization, and convergence remained the same, constraining model complexity by computational stability rather than statistical significance. For O_2_Hb and HHb, initial model specification included the fixed-effects structure Occlusion × Tracking × Test + Hemi, and the random-effects structure (1 + Occlusion + Tracking + Test | Subject) + (1 | Subject : Channel). For local efficiency, the fixed-effects structure was changed to Occlusion × Tracking × Test + ROI in order to reflect cortical organisation. For measures of eye movement and global efficiency, the fixed-effects structure excluded Hemi and ROI, and the random-effects excluded Channel. For hand velocity, neither Hemi, ROI, Tracking or Channel could be included in the fixed and/or random effects structure. Using Restricted Maximum Likelihood (REML), these initial model specifications did not converge or produce non-singular fits. Following an iterative simplification process, the most complex random-effects structure that was computationally stable across all measures of cortical activity and local efficiency was (1 | Subject) + (1 | Subject : Channel), and for all measures of behaviour and global efficiency it was (1 | Subject). Maintaining this random-effects structure, the initial fixed-effects specification was refit using Maximum Likelihood (ML). This approach returned the maximal converging model with standardise random-effects and fixed-effects structures. Fit of the accepted model was determined using conditional and marginal R2 (piecewiseSEM v2.3.0). To control Type 1 error, FDR correction was applied to all main and interaction effects for each family of tests per chromophore (O2Hb, HHb), for the family of Graph metrics (Local Efficiency, Global Efficiency) and the family of behavioural measures (Eye Velocity, Saccadic Displacement, Hand Velocity). Fixed interaction effects at *p* ≤ 0.05 were further analysed using Bonferroni pairwise correction (EMMEANS package v1.7.2). For brevity and clarity, the presentation of results is focused on the significant interaction effects that were most consistent in the behavioural and neuroimaging data. Estimated marginal means for significant pairwise comparisons (*p* ≤ 0.05) are also reported in the text. For additional details on the significance of fixed-effects for each dependent variable, the reader is directed to Tables 1, 2, 3 and 4.

**Table 3:** Summary of statistical results for local efficiency and global efficiency. Columns show: Graph Metric, Model, Effect, Chisq, degrees of freedom (df), P-values (raw and adjusted), conditional and marginal R2.

Graph metric	Model	Effect	Chisq	Df	*P* value	Adjusted *P* value	R2C	R2M
Local Efficiency	Data ~ Occlusion*Test*Tracking + ROI (1 | Subject) + (1 | Channel)	Occlusion	1.52	1	0.22	0.38	0.10	0.02
		Test	13.36	1	< 0.001 ***	< 0.01 **		
		Tracking	1.44	1	0.23	0.38		
		ROI	233.76	13	< 0.001 ***	< 0.001 ***		
		Occlusion: Test	2.04	1	0.15	0.38		
		Occlusion: Tracking	0.74	1	0.39	0.53		
		Test: Tracking	35.83	1	< 0.001 ***	< 0.001 ***		
		Occlusion: Test: Tracking	5.84	1	< 0.05 *	0.06		
Global Efficiency	Data ~ Occlusion*Test*Tracking+ (1 | Subject)	Occlusion	0.00	1	0.99	0.99	0.14	0.01
		Test	1.04	1	0.31	0.46		
		Tracking	0.03	1	0.86	0.92		
		Occlusion: Test	0.34	1	0.56	0.65		
		Occlusion: Tracking	0.53	1	0.47	0.58		
		Test: Tracking	2.45	1	0.12	0.35		
		Occlusion: Test: Tracking	1.59	1	0.21	0.38		

Significant values are in [bold].

**Table 4:** Summary of subsidiary analysis for eye velocity. Columns show: Model, Effect, Chisq, degrees of freedom (df), raw P-values, conditional and marginal R2.

Model	Effect	Chisq	Df	*P* value	R2C	R2M
Data ~ HVc * Occlusion * Test + (1 | Subject)	HVc	7.89	1	< 0.005 ***	0.92	0.89
	Occlusion	3050.08	1	< 0.001 ***		
	Test	0.77	1	0.3790		
	HVc: Occlusion	0.05	1	0.8325		
	HVc: Test	0.11	1	0.7417		
	Occlusion: Test	0.11	1	0.7385		
	HVc: Occlusion: Test	0.16	1	0.6913		

Significant values are in [bold].

## Data Availability

The datasets generated and analysed during the current study are available from the corresponding author on reasonable request.
